# Empiric broad-spectrum antibiotic therapy of nosocomial pneumonia in the intensive care unit: a prospective observational study

**DOI:** 10.1186/cc4919

**Published:** 2006-05-16

**Authors:** Francisco Álvarez-Lerma, Bernabe Alvarez, Pilar Luque, Francisco Ruiz, Jose-Maria Dominguez-Roldan, Elisabet Quintana, Cesar Sanz-Rodriguez

**Affiliations:** 1 Servicio de Medicina Intensiva, Hospital del Mar, Barcelona, Spain; 2 Servicio de Medicina Intensiva, Hospital General Universitario, Alicante, Spain; 3 Servicio de Medicina Intensiva, Hospital Clínico Universitario, Zaragoza, Spain; 4 Servicio de Medicina Intensiva, Complejo Hospitalario de Jaén, Spain; 5 Servicio de Medicina Intensiva, Hospital Universitario Virgen del Rocío, Sevilla, Spain; 6 Servicio de Medicina Intensiva, Hospital de la Santa Creu i Sant Pau, Barcelona, Spain; 7 Department of Clinical Research, Merck Sharp & Dohme of Spain, Madrid, Spain

## Abstract

**Introduction:**

Antibiotic de-escalation, which consists of the initial institution of empiric broad-spectrum antibiotics followed by antibiotic streamlining driven by microbiological documentation, is thought to provide maximum benefit for the individual patient, while reducing the selection pressure for resistance.

**Methods:**

To assess a carbapenem-based de-escalating strategy in nosocomial pneumonia (NP), a prospective observational study was conducted in critically ill patients with NP treated empirically with imipenem ± aminoglycoside/glycopeptide in 24 intensive care units of Spanish general hospitals. Overall, 244 patients were assessable (91% with late-onset NP). The primary outcome was therapeutic success 7–9 days post therapy.

**Results:**

Microbial identification – based on cultures of tracheal aspirates in 82% of patients, cultures of protected specimen brush in 33%, and cultures of bronchoalveolar lavage in 4% – was only available for 131 (54%) patients. Initial antibiotics were inadequate for 23 (9%) patients. Of the remaining patients, antibiotics were streamlined in 56 (23%) patients and remained unchanged in 14 (6%) patients based on microbiology data, in 38 (16%) patients despite microbiology data favouring de-escalation, and in 113 (46%) patients due to unknown aetiology. Overall, de-escalation was implemented in only 23% of patients with potentially multiresistant pathogens, compared with 68% of patients with the remaining pathogens (*P *< 0.001). Response rates were 53% for patients continuously treated with imipenem-based regimens and 50% for the de-escalated patients. Higher Acute Physiology, Age, and Chronic Health Evaluation II scores were associated with greater mortality, whereas adequate empiric antibiotic therapy protected against fatal outcomes. No increase of superinfection rates caused by emerging pathogens was observed. The costs associated with de-escalation were mainly dependent on the duration of hospitalization.

**Conclusion:**

This study mainly highlights the current practice of a specific algorithm of de-escalation solely based on the available microbiological data, and highlights the barriers to using it more widely. In this setting, de-escalation was less likely to occur in the presence of potentially multiresistant pathogens. Prior antibiotic administration and the low use of bronchoscopic techniques may have influenced negatively the implementation of de-escalation. Optimization of de-escalation strategies for NP should rely on a correct choice of empiric antibiotics, on appropriate microbiological investigations, and on a balanced interpretation of microbiological and clinical data.

## Introduction

Inadequate antibiotic treatment is a major risk factor for nosocomial pneumonia (NP)-attributed mortality [1-8] and is often associated with antibiotic-resistant Gram-negative bacteria and methicillin-resistant *Staphylococcus aureus *(MRSA) [1,3,4,9]. Changes of antibiotics often follow the isolation of microorganisms not covered by initial empiric antibiotics [4]. Yet the benefit of adequate antibiotic therapy may vanish if the onset of effective treatment is delayed [2,3,10].

De-escalation, which consists of the initial institution in severely ill patients of broad-spectrum antibiotics covering the most probable causative pathogens followed by antibiotic streamlining driven by microbiological documentation, is thought to provide maximum benefit for the individual patient, while reducing the selection pressure for resistance [11,12]. There are concerns, however, about the emergence of multidrug-resistant pathogens prompted by delayed streamlining of unnecessary antibiotics and the potential negative influence on diagnostic accuracy and economic impact of this strategy. De-escalation was the main cause of antibiotic modification in a series of patients with ventilator-associated pneumonia (VAP) from one institution [13], being more feasible in early-onset pneumonia and less frequent in the presence of nonfermenting Gram-negative bacilli (NFGNB) [13].

We designed a prospective multicentre observational study – Analysis of Antibiotic De-escalation for Nosocomial Pneumonia (ADANN) – to assess the clinical, microbiological, and pharmacoeconomic features of a carbapenem-based de-escalating strategy in intensive care unit (ICU) patients with NP. Our hypothesis was that de-escalation was feasible, yet highly dependent on the quality of microbiological investigations and the patient's clinical characteristics.

## Materials and methods

### Study design

This prospective, observational study was conducted in 24 ICUs of Spanish general hospitals in accordance with local regulations. The protocol was approved by Hospital del Mar's Ethical Committee.

Eligible patients aged ≥18 years had NP, were to receive empirically imipenem-based antibiotic regimens, and were required to have blood cultures and respiratory sampling performed before starting the study therapy. Written informed consent was obtained from the patients. Other diagnostic procedures were performed as needed.

Exclusion criteria included previous carbapenem administration during the ongoing NP episode; kidney dialysis, serum creatinine >267 μmol/l or creatinine clearance <20 ml/minute; development of pneumonia on days 1–4 post admission without concomitant risk factors (prior antibiotics/steroids, insulin-dependent diabetes mellitus, chronic obstructive pulmonary disease, chronic atelectasis, cirrhosis, active cancer); carbapenem hypersensitivity; expected survival <48 hours; pregnancy/nursing; and human immunodeficiency virus infection.

### Clinical management and study drug dosing regimen

The only therapeutic, diagnostic, or monitoring interventions allowed were those considered standard clinical practice. Visit 1 corresponded to the onset of study therapy. On visit 2 (3–5 days later), investigators streamlined antibiotics if favourable microbiological data were available. Patients were re-evaluated after discontinuation of therapy (visit 3) and again 7–9 days later (visit 4).

Antibiotics were generally dosed as recommended in the corresponding package inserts. The most common dose of imipenem was 1 g/8 hours daily. Patients with suspected *Pseudomonas *infection also received amikacin (15 mg/kg/day) or tobramycin (5 mg/kg/day). If MRSA infection was suspected, vancomycin (1 g/12 hours) or teicoplanin (400 mg/12-hour load, 400 mg/day thereafter) were added.

### Definitions

NP was defined per the Center for Disease Control and Prevention clinical criteria [14]. The bacteriologic diagnosis required one or more of the following criteria: tracheal aspirate (TA) cultures yielding ≥10^5 ^colony-forming units/ml; protected specimen brush cultures yielding ≥10^3 ^colony-forming units/ml; bronchoalveolar lavage (BAL) cultures yielding ≥10^4 ^colony-forming units/ml; blood or pleural fluid cultures yielding the same pathogen as respiratory samples; histopathologic evidence of pneumonia; or positive serology/identification of *Legionella pneumophila*.

The proposed de-escalation approach encompassed two stages. The first stage consisted of administering broad-spectrum empiric antibiotics (imipenem ± aminoglycoside and/or glycopeptide). Empiric therapy was considered adequate if ≥1 antibiotic to which all isolates were susceptible was used after the initial respiratory sampling. Carbapenem-resistant, aminoglycoside-susceptible *Pseudomonas *infections were always considered inadequately treated, even if they were treated with imipenem in combination with an active aminoglycoside. The second stage involved antibiotic streamlining based on available microbiological data. The investigators were provided with no rules and with no guidance for antibiotic de-escalation. Streamlining was considered adequate if the empiric regimen was changed to piperacillin/tazobactam, or to an anti-pseudomonal cephalosporin if susceptible *Pseudomonas aeruginosa *was isolated, or to a non-antipseudomonal β-lactam (witholding aminoglycosides and/or glycopeptides if possible) if NFGNB were absent. Three independent reviewers judged the adequacy of the empiric therapy and the correctness of de-escalation.

Clinical success required complete resolution of all attributable symptoms, signs, and radiographic and laboratory abnormalities. Failures included unsatisfactory clinical response, any antibiotic addition or substitution, or death of any cause. Deaths were attributed to NP if they occurred before any objective response to antimicrobials or if NP contributed to death in patients with comorbidities. The judgement of attributable mortality was made by the investigator who followed up the patient at each study site.

### Statistics

The primary outcome was therapeutic success 7–9 days post study therapy, which was estimated at ~70% for sample size calculations. Using a significance level of 0.05, a power of 94%, and 10% of patients lost to follow-up, 247 patients were required for the 95% confidence interval of the response rate to fall between 63% and 76%. Preplanned secondary objectives included descriptive and comparative effectiveness, toxicity, mortality, superinfections, and cost analyses.

Effectiveness analyses were performed on modified intention-to-treat (MITT) and patient-evaluable (PE) populations. The MITT population only excluded patients misdiagnosed or dying before treatment began. PE patients met MITT criteria, underwent follow-up until visit 4 or death and had sufficient information to determine outcomes, and received study therapy for ≥7 days for cured cases and for ≥3 days for failures.

Chi-square tests were used for effectiveness and mortality contingency analyses. Forward stepwise logistic regression analysis with a cutoff *P *value of 0.05 was used to determine the relationship between outcome variables and independent variables previously identified in univariate analyses (*P *< 0.05). For the costs, chi-square and Kruskal–Wallis tests were used for categorical variables and continuous variables, respectively.

## Results

**Figure 1:**
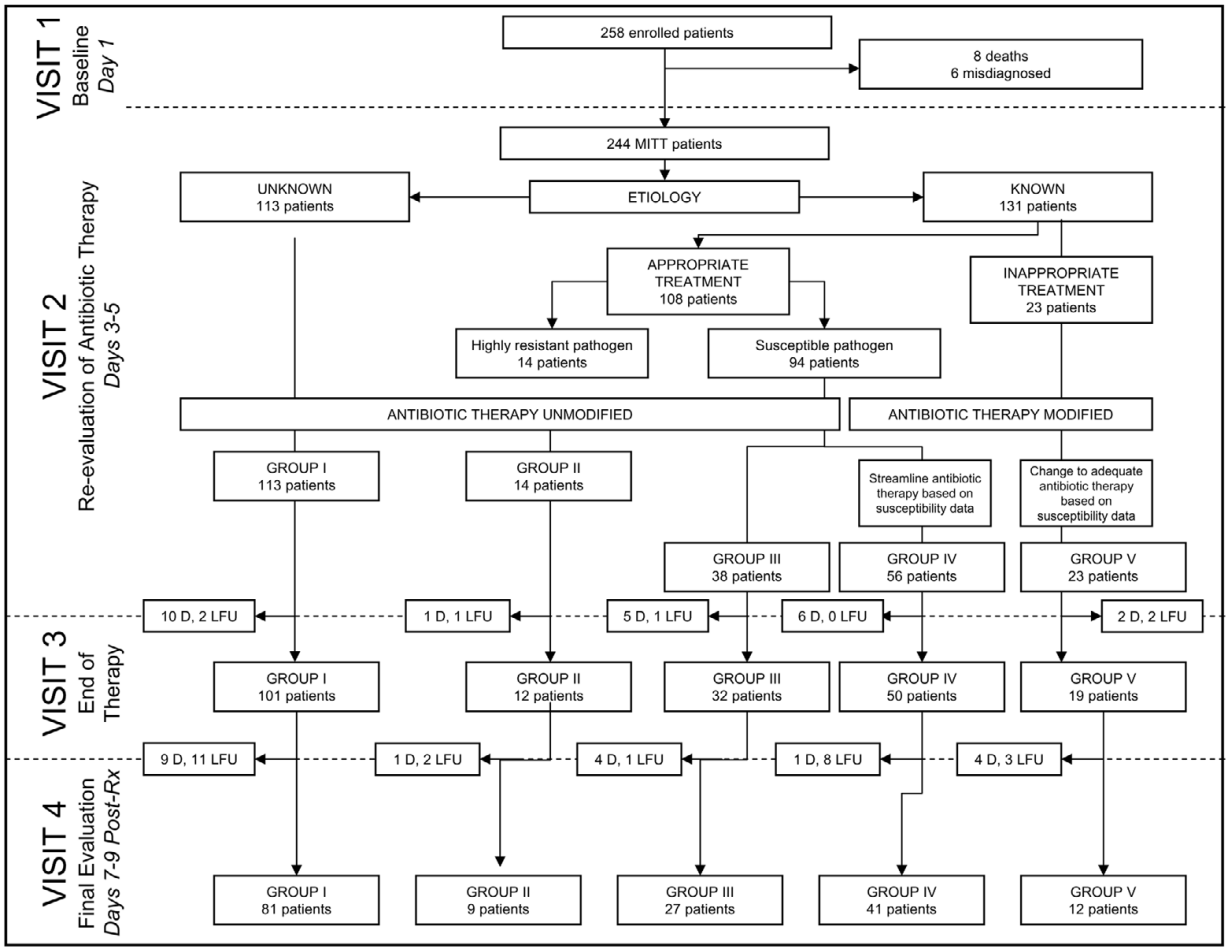
Study overview: flow chart representing the patient flow throughout the different study visits. Visit 1, onset of study therapy; visit 2 (3–5 days later), investigators streamlined antibiotics if favourable microbiological data were available; visit 3, patients were re-evaluated after discontinuation of therapy, visit 4, patients were re-evaluated 7–9 days later. MITT, modified intention-to-treat; D, dead; LFU, lost to follow-up.

Two hundred and fifty-eight patients with a first episode of NP were enrolled from April 2000 to June 2001. Fourteen patients did not meet the MITT criteria and 31 patients were lost to follow-up (Figure 1). No selection bias was identified in a comparative analysis of the baseline characteristics of patients lost to follow-up. Overall, there were 244 MITT patients and 213 PE patients (170 alive on visit 4, 43 dead between visits 2 and 4) (Figure 1). Tables 1 and 2 display patient demographics and the empiric antibiotic therapy, respectively.

Favourable response rates among MITT patients were 75.4% upon completion of therapy and were 50.4% 7–9 days later, with no significant differences across treatment groups (Table 3). The PE analysis was supportive of the primary MITT analysis. Increasing baseline Acute Physiology, Age, and Chronic Health Evaluation II scores were associated with a lower likelihood of favourable response (*P *< 0.01). The mortality analysis was performed only in PE patients. Overall, 20.2% PE patients died. The NP-attributed mortality was 13.6%, which represented 67.4% of all deaths.

Microbiological identification was based on TA cultures performed in 199 (81.6%) patients, protected specimen brush cultures performed in 80 (32.8%) patients, and BAL cultures performed in only 10 (4.1%) patients. On visit 2, bacteriologic documentation was only available for 131 (53.7%) patients (Table 4). NFGNB (mainly *Pseudomonas *and *Acinetobacter*), Enterobacteriaceae, and *S. aureus *represented 38.4%, 20.5%, and 17.8% of all pathogens causing monomicrobial infections, respectively. NFGNB and *S. aureus *were also common in polymicrobial infections and occurred more frequently in patients previously treated with antibiotics (*P *= 0.02 for monomicrobial infections, *P *< 0.001 for polymicrobial infections). On visit 2, patients were grouped based on the availability of microbiological data and therapeutic decisions as follows: Group I included 113 (46.3%) patients with an unknown aetiology and unmodified therapy; Group II included 14 (5.7%) patients with resistant organisms, who had unmodified therapy; Group III included 38 (15.6%) patients with susceptible organisms, who had unmodified therapy; Group IV included 56 (23.0%) patients who had susceptible organisms and whose therapy was modified accordingly; and Group V included 23 (9.4%) patients who initially received inadequate antibiotic therapy, which was later modified on the basis of cultures.

For patients included in the latter group, bacteriologic investigations yielded carbapenem-resistant *Acinetobacter *spp. (seven patients); *P. aeruginosa *(three patients) and *Pseudomonas putida *(one patient); *Escherichia coli *(one patient), *Proteus mirabilis *(one patient), and *Serratia marcescens *(one patient) with full or intermediate imipenem resistance; MRSA (seven patients, none received empiric glycopeptides); *Mycobacterium tuberculosis *(two patients); and *Candida albicans *(one blood culture isolate). Both the overall mortality and the NP-attributed mortality were higher in inadequately treated patients despite modification of initial antibiotics within 72 hours. A logistic regression analysis confirmed the association between inadequate therapy and overall mortality (Table 3).

Susceptible strains were identified in 108 (44.3%) patients, yet antibiotics were only streamlined in 56 patients (Group IV). The overall de-escalation rate was therefore 23.0% (56/244 patients). This proportion increased to 25.3% (56/221 patients) when inadequate treatments were excluded, to 42.7% (56/131 patients) when episodes of unknown aetiology were excluded, and to 51.9% (56/108 patients) when episodes with susceptible strains only were considered. Empiric antibiotics remained unchanged in 52 (21.3%) patients with documented aetiology.

Fourteen patients did not have narrower spectrum alternatives (Group II). Pathogens in this treatment group included *Acinetobacter *spp. (11 cases), *P. aeruginosa *(two cases), *Klebsiella pneumoniae *(one case), and *Enterobacter aerogenes *(one case). Empiric antibiotics were not streamlined despite favourable microbiological data in the remaining 38 patients (Group III). Overall, antibiotics were streamlined in only nine of 39 patients (23.1%) with potentially multiresistant pathogens (for example, NFGNB and MRSA), compared with 47 of 69 patients (68.1%) among the remaining pathogens (*P *< 0.001). Of the 30 patients with potentially multiresistant pathogens who were not de-escalated, 14 belonged in Group II and 16 in Group III. Finally, the initial empiric regimen was also maintained in 113 (46.3%) patients with unknown aetiology (Group I).

The duration of antibiotic therapy was similar in patients who were de-escalated (median, 18 days; range, 4–55 days) versus that in patients who were not de-escalated (median, 16 days; range, 3–65 days; *P *> 0.05), yet was longer than for patients with unknown aetiology (median, 13 days; range, 4–72 days; *P *= 0.02). Not surprisingly, imipenem was less used in the former (median, 5 days versus 14 days versus 11 days; *P *< 0.001). Superinfections were diagnosed in 16 (6.6%) patients, most of whom had prior antibiotic exposure. Bacteriologic testing of TA (12 patients), protected specimen brush (one patient), and BAL (one patient) yielded *P. aeruginosa *(six cases), *Acinetobacter baumannii *(four cases), MRSA (three cases), methicillin-susceptible *S. aureus *(one case), *Klebsiella oxytoca *(one case), and *Enterobacter cloacae *(one case). Two additional patients had positive blood cultures to *C. albicans *and *Enterococcus faecalis*, respectively. Interestingly, superinfection rates were similar across treatment groups (Group I, 7.1%; Group II, 7.1%; Group III, 2.6%; Group IV, 5.4%; Group V, 13.0%) regardless of the different duration of broad-spectrum therapy.

Antibiotic-related adverse reactions included seizures (three episodes probably or possibly related to imipenem), thrombocytosis (one episode probably related to cefotaxime), rash (one episode probably related to vancomycin), acute tubular necrosis (one episode possibly related to amikacin), and elevated serum aminotransferase levels (one episode possibly related to imipenem). Adverse reactions were generally mild to moderate, only causing therapy discontinuation in three patients (two seizures, one rash).

A cost analysis focusing on the duration and costs of hospitalization was conducted from Spanish National Health Insurance perspective (Table 5). The duration and costs of the ICU stay were higher for patients with microbiological diagnosis who were not de-escalated (*P *< 0.001 and *P *= 0.001, respectively), while the costs of diagnostics and drugs other than antibiotics were higher for inadequately treated patients and for patients not de-escalated despite favourable microbiology (*P *= 0.04). Although this resulted in significant differences in total cost per NP episode (*P *= 0.01), there were no differences in the total daily costs between treatment groups, which averaged €326.07 (*P *= 0.35).

## Discussion

In this study, initial empiric antibiotics were streamlined in 51.9% episodes with susceptible microbial isolates in patients who were given appropriate initial therapy. This proportion fell to 42.7% when episodes of unknown aetiology were excluded, to 25.3% when inadequately treated patients were excluded, and to 23.0% when all 244 evaluable patients were considered. These figures are in general agreement with an earlier report [13], where de-escalation was possible in 38% of VAP episodes with susceptible pathogens (34.2% and 31% when resistant strains and unknown aetiology were included, respectively). These data are, however, much higher than the 6.1% found in a previous cohort of patients with VAP [1]. The low de-escalation rate in this latter experience was probably due to the high prevalence of *Pseudomonas *(48.3%), much greater than that found in our study (30.6% (33/108 patients)) and than that recently reported by Rello and colleagues (17.4%) [13]. The impact of local susceptibility patterns on the de-escalation strategy is further evidenced by our observation that patients with potentially multiresistant pathogens (NFGNB and MRSA) were de-escalated less frequently than patients with other pathogens (23.1% versus 68.1%; *P *≤ 0.001).

The feasibility of de-escalation may also depend on the patients' clinical characteristics. The lower de-escalation rate in our study compared with that previously reported in VAP patients [13] could be partly due to the intensive prior use of antibiotics (79.1%) in our series and the larger proportion of late-onset episodes in our study (90.6% versus 62.8%). Also, in our study de-escalation was not implemented in 15.6% of all patients with known aetiology. Similarly, Rello and colleagues [13] reported that empirical therapy remained unchanged in 10% of VAP patients due to delayed clinical resolution.

De-escalation was not carried out in 113 patients with negative cultures, which resulted in a prolonged administration of imipenem (median, 11 days). In our view, a wider use of the bronchoscopic techniques, with higher specificity than TA cultures, may have facilitated the proposed de-escalation strategy in our study. Although we believe the high proportion of patients with negative cultures who were not de-escalated was probably influenced by the lack of specific recommendations for de-escalation in this patient group, whether or not de-escalation was still possible remains an issue to be defined. In Rello and colleagues' study [13], patients with unknown aetiology were re-evaluated 2–3 days after the onset of therapy; those clinically improved continued to receive short-course antibiotics, while nonresponders were switched to different antibiotics. The authors concluded that de-escalation was not possible in patients with negative cultures since antibiotics were only changed in 30% of the episodes due to clinical deterioration. The results of a more recent prospective study, however, suggest that patients with a clinical suspicion of VAP and culture-negative BAL can have empiric antimicrobial therapy safely discontinued within 72 hours or in some cases withheld altogether [15].

De-escalation is expected to minimize inappropriate initial antibiotic therapies, which result in higher mortality [1-8]. In our study, inadequate initial empiric therapy occurred in 9.4% of MITT patients and was associated with increased mortality (Table 3). This rate was similar to that recently reported in VAP patients [13]. Pathogens identified in this patient group included carbapenem-resistant *Acinetobacter *spp., MRSA (only in patients not given empiric glycopeptides), *M. tuberculosis*, and *C. albicans*. The question remains of how many of these patients could have benefited from an appropriate empiric antibiotic regimen, had they been previously identified as being at high risk for antibacterial-resistant infections.

While it has been shown that extensive carbapenem use may lead to increases in resistance [16,17], in some studies imipenem-based empiric strategies did not lead to higher rates of bacterial and fungal superinfections [18,19]. Yet, it is generally agreed that in order to avoid unnecessary selection pressure on microorganisms resulting from the use of broad-spectrum antibiotics [20-23], carbapenems should always be used for empiric therapy based on local epidemiology. In order to control potential confusing factors related to the different trends of antibiotic use across participating institutions, the study design only allowed the enrolment of patients empirically treated with imipenem-based regimens, which are common in Spanish institutions based on the local pathogens and antibiotic susceptibilities [24,25]. Interestingly, the incidence of superinfections was low despite the large proportion of patients (67.6%) continuously treated with imipenem until clinical resolution. Nonetheless, no general conclusion can be drawn regarding the impact of a carbapenem-based de-escalation strategy on the emergence of resistant pathogens based on this study, since the proportion of NP episodes empirically treated with imipenem varied significantly between institutions.

The cost analysis suggested that resource use associated with de-escalation for NP was mainly dependent on the costs of diagnostic and therapeutic procedures other than antibiotics and, probably, the duration of hospitalization (probably driven by the severity of disease), but not heavily influenced by antibiotics acquisition costs. These data challenge the assumption that the widespread use of broad-spectrum antibiotics in patients lacking a definite microbiologic diagnosis may escalate costs.

This observational study has several limitations; most notably, the lack of a control group and pre-established de-escalation recommendations, and the study focus being on patients empirically treated with imipenem-based regimens only. The study design was therefore not appropriate to draw any conclusions regarding the benefits of carbapenem-based de-escalation strategies. Another major limitation was the large proportion of patients lacking microbiological documentation in the absence of guidance on the duration of empiric antibiotic therapy. Changes of therapy in this patient group were most probably prompted by nonresponse to empiric antibiotics. Finally, there were no standardized criteria for admission, diagnosis, and the initial choice of empiric antibiotics across participating ICUs. The relatively low cure rates observed could be partly due to suboptimal dosing of the different antibiotics used for empiric therapy resulting from the wide pharmacokinetic variations typically occurring in critically ill patients. Further studies are needed to evaluate the magnitude of changes in antibiotic use and clinical outcomes resulting from the implementation of de-escalation strategies.

## Conclusion

The findings herein reported were based on a diagnostic strategy mainly supported by TA cultures obtained before the initiation of empiric imipenem-based therapy in a cohort of patients with 90% of late-onset NP episodes. De-escalation was implemented in 51.9% of episodes with susceptible microbial isolates but was not performed if the pathogen remained unknown. The intensive prior use of antibiotics and the infrequent use of invasive bronchoscopic techniques may have contributed to the large number of episodes with unknown aetiology. De-escalation was more likely to occur in the presence of potentially multiresistant pathogens, mainly NFGNB.

The results of this study suggest that optimization of de-escalation strategies for the treatment of critically ill patients with NP should rely on the following principles. First, the choice of empiric antibiotics should be based both on local pathogen prevalence and antibacterial susceptibility and on the identification of patients with selected clinical parameters at high risk of developing infections caused by multi-resistant microorganisms. Good-quality culture sampling is also needed before starting antibacterial therapy. Finally, specific recommendations need be developed for patients with unknown aetiology in order to reduce the duration of empiric therapy.

## Key messages

• 	Inadequate empirical antibiotic therapy contributes substantially to attributed mortality in critically ill patients with NP. Appropriate antibiotic therapy for NP continues to be a major challenge in the ICU setting.

• 	The antibiotic de-escalation strategy has recently been introduced to increase coverage against the most frequently causative pathogens, including multiresistant strains, and to reduce the selection pressure for antimicrobial resistance.

• 	In a prospective observational multicentre study, the causative organism was only identified in 54% of patients with NP. De-escalation was used in 23% of cases. In 16% of cases, despite microbiology data favouring de-escalation, it was not applied. Empirical antibiotic treatment was inadequate in 9% of patients.

• 	Response rates were 53% for patients continuously treated with imipenem-based regimens versus 50% for those patients who were de-escalated. Patients with inappropriate empirical treatment had significantly higher mortality.

## Abbreviations

ADANN = Analysis of Antibiotic De-escalation for Nosocomial Pneumonia; BAL = bronchoalveolar lavage; ICU = intensive care unit; MITT = modified intention-to-treat; MRSA = methicillin-resistant *Staphylococcus aureus*; NFGNB = nonfermenting Gram-negative bacilli; NP = nosocomial pneumonia; PE = patient-evaluable; TA = Tracheal aspirate; VAP = ventilator-associated pneumonia.

## Competing interests

This observational study was sponsored by Merck Sharp & Dohme of Spain. CS-R is an employee of Merck Sharp & Dohme of Spain and holds stock options in the Company. FA-L, BA, PL, FR, J-MD-R, and EQ have no financial or non-financial competing interests to be disclosed.

## Authors' contributions

FA-L made substantial contributions to the conception and design of the study, served as the Clinical Monitor for the study, participated in the analysis and interpretation of the data, and was involved in the drafting of the manuscript. CS-R contributed to the analysis and interpretation of data, and was involved in the drafting of the manuscript. BA, PL, FR, J-MD-R, and EQ were involved in revising the manuscript critically. All authors read and approved the final manuscript.

## Figures and Tables

**Table 1:** Baseline characteristics of modified intention-to-treat patients grouped as defined per bacteriologic documentation (visit 2)

	Group I (*n *= 113, 46.3%)	Group II (*n *= 14, 5.7%)	Group III (*n *= 38, 15.6%)	Group IV (*n *= 56, 23.0%)	Group V (*n *= 23, 9.4%)	Overall (*n *= 244, 100%)
Gender (%)						
Male	75.2	78.6	68.4	78.6	73.9	75.0
Female	24.8	21.4	31.6	21.4	26.1	25.0
Age (years)						
Mean (standard deviation)	54.8 (16.6)	54.9 (22.9)	55.5 (20.1)	52.8 (18.7)	51.3 (22.4)	54.1 (18.5)
Diagnosis of nosocomial pneumonia (%)						
Early onset	8.0	-	7.9	14.3	13.0	9.4
Late onset	92.0	100	92.1	85.7	87.0	90.6
Prior therapy (%)						
Antibiotics	86.7	85.7	76.3	64.3	78.3	79.1
Steroids	19.5	21.4	26.3	21.4	21.7	21.3
Vital signs (%)						
Fever (>38.2°C)	50.4	42.9	71.1	55.4	39.1	53.3
Tachypnea (>20 cycles/minute)	36.6	35.7	34.2	39.3	40.9	37.2
Systolic hypotension (<100 mmHg)	37.2	57.1	47.4	33.9	43.5	39.8
Tachycardia (>110 beats/minute)	30.4	35.7	23.7	28.6	30.4	29.2
Pulmonary function (%)						
PaO_2_/FiO_2 _< 250 mmHg^a^	85.2	81.8	89.3	83.0	50.0	81.5
Other therapies (%)						
Mechanical ventilation^b^	76.1	85.7	92.1	87.3	95.5	83.5
Vasoactive drugs	45.5	50.0	45.9	33.9	54.5	44.0
APACHE II score						
Mean (standard deviation)	17.4 (7.0)	17.6 (9.0)	17.3 (8.3)	16.0 (6.0)	18.3 (7.8)	17.2 (7.2)
0–8 (%)	8.0	14.3	15.8	8.9	4.3	9.4
9–16 (%)	42.5	28.6	31.6	42.9	34.8	39.3
17–24 (%)	33.6	28.6	39.5	42.9	43.5	37.3
>24 (%)	15.9	28.6	13.2	5.4	17.4	13.9
Concomitant diseases (%)						
Any	67.3	64.3	47.4	58.9	60.9	61.5
Cardiovascular disease	25.7	42.9	21.1	21.4	30.4	25.4
Chronic respiratory disease	25.7	21.4	10.5	17.9	21.7	20.9
Liver disease	8.0	7.1	5.3	7.1	-	6.6
Renal dysfunction	5.3	7.1	2.6	3.6	-	4.1
Active neoplasia	4.4	14.3	2.6	8.9	17.4	7.0

Group I, patients with an unknown aetiology and unmodified therapy; Group II, patients with resistant organisms, who had unmodified therapy; Group III, patients with susceptible organisms, who had unmodified therapy; Group IV, patients who had susceptible organisms and whose therapy was modified accordingly; and Group V, patients who initially received inadequate antibiotic therapy, which was later modified on the basis of cultures. APACHE, Acute Physiology, Age, and Chronic Health Evaluation. ^a^Chi-square test, *P *< 0.01. ^b^Chi-square test, *P *= 0.052.

**Table 2:** Initial empiric antibiotic therapy in modified intention-to-treat patients grouped as defined per bacteriologic documentation (visit 2)

	Group I (*n *= 113, 46.3%)	Group II (*n *= 14, 5.7%)	Group III (*n *= 38, 15.6%)	Group IV (*n *= 56, 23.0%)	Group V (*n *= 23, 9.4%)	Overall (*n *= 244, 100%)	Chi-square *P *value
Dose of imipenem (%)							0.51
<3 g/day	19.5	-	10.5	9.0	17.3	14.3	
3 g/day	62.8	85.7	73.7	60.7	65.2	65.6	
>3 g/day	17.7	14.3	15.8	30.4	17.4	20.1	
Empiric regimen (%)							0.64
Imipenem only	19.5	7.1	18.4	21.4	8.7	18.0	
Imipenem + aminoglycoside	37.2	50.0	42.2	48.2	34.8	41.0	
Amikacin	24.8	28.6	21.1	41.1	26.1	28.3	
Tobramycin	12.4	21.4	21.1	7.1	8.7	12.7	
Imipenem + glycopeptide	15.0	21.4	21.1	8.9	21.7	15.6	
Vancomycin	9.7	21.4	13.2	8.9	13.0	11.1	
Teicoplanin	5.3	-	7.9	-	8.7	4.5	
Imipenem + aminoglycoside + glycopeptide	28.3	21.4	18.4	21.4	34.8	25.4	

Group I, patients with an unknown aetiology and unmodified therapy; Group II, patients with resistant organisms, who had unmodified therapy; Group III, patients with susceptible organisms, who had unmodified therapy; Group IV, patients who had susceptible organisms and whose therapy was modified accordingly; and, Group V, patients who initially received inadequate antibiotic therapy, which was later modified on the basis of cultures.

**Table 3:** Effectiveness and mortality analyses for each treatment group as defined per bacteriologic documentation (visit 2)

	Group I	Group II	Group III	Group IV	Group V	Overall
**Effectiveness response rates**						
Modified intention-to-treat population	(*n *= 113)	(*n *= 14)	(*n *= 38)	(*n *= 56)	(*n *= 23)	(*n *= 244)
End of therapy (visit 3) (%)	81.4	78.6	68.4	75.0	56.5	75.4
Final evaluation (visit 4) (%)	54.0	64.3	44.7	50.0	34.8	50.4
Patient-evaluable population	(*n *= 100)	(*n *= 11)	(*n *= 36)	(*n *= 48)	(*n *= 18)	(*n *= 213)
Final evaluation (visit 4) (%)	61.0	81.8	47.2	58.3	44.4	57.7
**Mortality rates**						
Patient-evaluable population	(*n *= 100)	(*n *= 11)	(*n *= 36)	(*n *= 48)	(*n *= 18)	(*n *= 213)
Overall mortality (%)	19.0	18.2	25.0	14.6	33.3	20.2
Nosocomial pneumonia-attributable mortality (%)	15.0	9.1	8.3	8.3	33.3	13.6

Variables independently associated with overall mortality by logistic regression analysis	Adjusted odds ratio		95% confidence interval		*P*	

Age (year of age)	1.019		1.001–1.038		0.033	
Baseline APACHE II score (per point)	1.064		1.019–1.111		0.004	
Treatment group (referred to Group V)					0.014	
Group I	0.341		0.118–0.859			
Group II	0.116		0.020–0.691			
Group III	0.341		0.108–1.080			
Group IV	0.138		0.042–0.454			
Constant	0.151		0.038–0.607		-	

Forward stepwise logistic regression analysis (cut-off *P *value of 0.05) was used to determine the relationship between mortality and independent baseline variables previously identified in univariate analyses (*P *< 0.05), including: age, mechanical ventilation, Acute Physiology, Age, and Chronic Health Evaluation (APACHE) II score, treatment group, and adequacy of initial empiric therapy. Group I, patients with an unknown aetiology and unmodified therapy; Group II, patients with resistant organisms, who had unmodified therapy; Group III, patients with susceptible organisms, who had unmodified therapy; Group IV, patients who had susceptible organisms and whose therapy was modified accordingly; and Group V, patients who initially received inadequate antibiotic therapy, which was later modified on the basis of cultures.

**Table 4:** Microbiologic documentation available in visit 2 in modified intention-to-treat patients with nosocomial pneumonia

	Prior antibiotic therapy		Overall	Percentage performed	Chi-square *P *value
				
	No	Yes			
Pathogens (%)					0.002
None	27.5	51.8	52.5		
Monomicrobial	33.3	29.0	29.9		
Polymicrobial	39.2	19.2	23.8		
Yield of microbial investigations (%)					
Tracheal aspirate	87.5	79.5	81.4	81.6	
Protected specimen brush	96.3	67.9	80.0	32.8	
Bronchoalveolar lavage	100	71.4	77.5	4.1	
Pathogens identified (all patients)					
Monomicrobial	17	56	73		0.02
*Pseudomonas aeruginosa*	1	13	14		
Other *Pseudomonas*	0	2	2		
*Acinetobacter baumannii*	0	11	11		
Other *Acinetobacter*	0	1	1		
*Enterobacteriaceae*	3	12	15		
*Staphylococcus aureus*	4	9	13		
*Haemophylus influenzae*	6	5	11		
*Streptococcus pneumoniae*	2	2	4		
*Streptococcus viridans *group	0	0	0		
Other	1	1	2		
Polymicrobial	20	38	58		<0.001
Mixed, including NFGNB and methicillin-resistant *S. aureus*	4	23	27		
*H. influenzae *plus *Streptococcus *spp.	9	2	11		
Other	7	13	20		
Pathogens identified (Group V^a^)					
Monomicrobial	1	7	8		
*P. aeruginosa*	1	2	3		
Other NFGNB	0	2	2		
*Enterobacteriaceae*	0	1	1		
Methicillin-resistant *S. aureus*	0	1	1		
Other	0	1	1		
Polymicrobial	4	11	15		
Mixed, including NFGNB and methicillin-resistant *S. aureus*	1	8	9		
Other	3	3	6		

NFGNB, nonfermenting Gram-negative bacilli. Overall, there were 33 *Pseudomonas *isolates, including 30 *Pseudomonas aeruginosa*, two *Pseudomonas putida*, and one *Pseudomonas *spp., and 22 *Acinetobacter *isolates, including 20 *A. baumannii*, one *Acinetobacter haemolyticus*, and one *Acinetobacter calcoaceticus*. ^a^Group V, patients who initially received inadequate antibiotic therapy, which was later modified on the basis of cultures.

**Table 5:** Cost analysis in modified intention-to-treat patients grouped as defined per bacteriologic documentation (visit 2)

	Group I (*n *= 113, 46.3%)	Group II (*n *= 14, 5.7%)	Group III (*n *= 38, 15.6%)	Group IV (*n *= 56, 23.0%)	Group V (*n *= 23, 9.4%)	Overall (*n *= 244, 100%)	Kruskal–Wallis *P *value
Hospital admission (days)	23.8 (39.9)	19.9 (19.9)	22.1 (36.0)	14.9 (19.0)	33.8 (81.2)	22.3 (40.9)	0.46
ICU admission (days)	20.5 (34.1)	34.0 (32.2)	36.7 (24.2)	23.7 (14.5)	17.8 (79.0)	24.3 (36.4)	<0.001
Costs of hospitalization in the general ward (€)	4,983.40 (8,754.20)	4,160.47 (4,461.11)	4,835.13 (8,023.24)	2,983.81 (4,164.14)	7,264.92 (8.754.20)	4,669.23 (8,971.35)	0.44
Costs of ICU admission (€)	6,725.70 (11,412.94)	11,469.22 (10,872.13)	12,055.11 (8,283.93)	7,860.99 (4,965.19)	5,749.28 (26,069.12)	7,996.38 (12,182.74)	0.001
Antibiotic acquisition costs (€)	539.98 (2,636.00)	533.03 (622.62)	487.49 (1,002.59)	322.06 (300.38)	360.09 (446.92)	464.44 (1,850.30)	0.97
Other therapeutic and diagnostic procedures (€)	631.26 (462.19)	591.61 (278.65)	853.59 (531.50)	652.41 (399.60)	855.75 (513.94)	689.63 (463.63)	0.04
Total costs per episode (€)	12,880.35 (9,420.68)	16,754.33 (10,977.94)	18,231.32 (13,402.92)	11,819.28 (7,007.02)	14,230.04 (10,571.25)	13,819.67 (10,041.06)	0.01
Total daily costs (€)	324.05 (102.29)	327.16 (41.17)	337.95 (46.27)	332.19 (39.40)	301.22 (156.78)	326.07 (89.22)	0.35

Data presented as mean (standard deviation). ICU, intensive care unit. Costs were estimated using drug label information and data published by Institut Municipal d'Assistència Sanitària and Laboratori de Referència de Catalunya (Barcelona, Spain) updated through 2001. Group I, patients with an unknown aetiology and unmodified therapy; Group II, patients with resistant organisms, who had unmodified therapy; Group III, patients with susceptible organisms, who had unmodified therapy; Group IV, patients who had susceptible organisms and whose therapy was modified accordingly; and Group V, patients who initially received inadequate antibiotic therapy, which was later modified on the basis of cultures.
